# Decision Making under Risk in Patients Suffering from Schizophrenia or Depression

**DOI:** 10.3390/brainsci11091178

**Published:** 2021-09-07

**Authors:** Theresa Benke, Josef Marksteiner, Beatrix Ruepp, Elisabeth M. Weiss, Laura Zamarian

**Affiliations:** 1 Department of Psychiatry and Psychotherapy A, Hall State Hospital, 6060 Hall in Tirol, Austria; theresa.benke@tirol-kliniken.at (T.B.); josef.marksteiner@tirol-kliniken.at (J.M.); beatrix.ruepp@tirol-kliniken.at (B.R.); 2 Department of Psychology, University of Innsbruck, 6020 Innsbruck, Austria; elisabeth.weiss@uibk.ac.at; 3 Department of Neurology, Medical University of Innsbruck, 6020 Innsbruck, Austria

**Keywords:** risk-taking, schizophrenia, major depression, cognitive flexibility, memory, executive functions

## Abstract

Studies have reported difficulties in decision making for patients with schizophrenia or depression. Here, we investigated whether there are differences between schizophrenia patients, depressed patients, and healthy individuals (HC) when decisions are to be made under risk and cognitive flexibility is required. We were also interested in the relationships between decision making, cognitive functioning, and disease severity. Thirty HC, 28 schizophrenia patients, and 28 depressed patients underwent structured clinical assessments and were assessed by the Positive and Negative Syndrome Scale or Hamilton Rating Scale. They performed the Probability-Associated Gambling (PAG) Task and a neuropsychological test battery. Both patient groups obtained lower scores than HC in memory and executive function measures. In the PAG task, relative to HC, depressed patients made slower decisions but showed a comparable number of advantageous decisions or strategy flexibility. Schizophrenia patients were slower, riskier, and less flexible compared to HC. For them, the decision making behavior correlated with the symptom severity. In both groups, decision making scores correlated with memory and executive function scores. Patients with schizophrenia or depression may have difficulties under risk when quick and flexible decisions are required. These difficulties may be more pronounced in patients who have marked cognitive deficits or severe clinical symptoms.

## 1. Introduction

Decision making is an essential ability to function in everyday life. It can critically influence one’s physical and psychic well-being, interpersonal functioning, financial, living, and job situation, and many more. Difficulties to make advantageous decisions may have a huge impact in the near but also the far future. Over the last years, this key function has increasingly become a focus of scientific research. Studies have regarded not only normal functioning but also the difficulties that, for example, neurological patients or patients with psychiatric conditions may encounter when making advantageous decisions [1,2,3,4]. Schizophrenia and major depression are two neuropsychiatric conditions that are marked, although to a different degree, by cognitive deficits, especially in attention, information processing, memory, and executive function [5,6,7,8,9,10,11]. Abnormalities in decision making under ambiguity have also been reported for both these conditions [12,13,14,15,16,17,18,19,20,21,22,23]. Very few investigations have focused on decision making under risk in patients with schizophrenia and patients with depression. However, no study thus far has assessed which of these two clinical groups might have more difficulties under identical decision making under risk conditions. The current study aimed to fill this gap and to investigate possible differences between schizophrenia patients, patients with major depression, and healthy individuals in decision making under risk by adopting the *Probability-Associated Gambling* task (PAG task). Furthermore, we were interested in possible associations between the decision making performance, selected cognitive functions, and clinical symptoms of schizophrenia and major depression.

Decision making is a complex ability that requires, among other processes, the evaluation of options, the formation of a preference, the execution and completion of an action, and the processing of the consequences [4,24,25,26,27]. In neuropsychological research, two types of decision situations are distinguished depending on the amount and type of information available: decisions under ambiguity and decisions under risk. In decision making under ambiguity, information about the options and consequences is missing or misleading, and one has to rely on feedback, experience, and emotional processing to learn which choice is the most advantageous [27,28]. Differently, in decision making under risk, information about the options and consequences is defined explicitly or is calculable/estimable [4,24,27]. In these situations, logical or mathematical deliberations, estimations, and executive function play a relevant role [24]. Patients with schizophrenia or major depression do not only show problems in emotional processing and emotional regulation but also have several cognitive deficits [29,30]. Different studies have reported cognitive impairments in attention, information processing, memory, and executive function for both patients with schizophrenia and patients with major depression [5,6,7,8,9,10,11,31]. In schizophrenia and major depression, cognitive deficits occur regardless of age, number of episodes, course of disease and severity, and often persist in asymptomatic phases in various forms [32,33,34,35,36]. Evidence about abnormalities in decision making for both neuropsychiatric conditions has also been reported.

Studies using the *Iowa Gambling Task* (*IGT*) [37] to investigate decision making under ambiguity have found that both patients with schizophrenia and patients with major depression significantly differ from healthy individuals [12,14,17,20,21,23,38]. In the IGT, participants are presented with four options but are not informed about the magnitude and probability of win/loss of each alternative. They have to learn from feedback which alternatives are more advantageous and adapt their strategy by “trial and error” to maximize their capital. Patients with schizophrenia and positive symptomatology show a similar decision pattern to that of patients with damage in the orbitofrontal cortex (OFC) by preferring risky options [39]. Their poorer performance on the IGT may be related to disorders in emotional processing, emotion regulation, and learning from experience [39,40]. Patients with depression also differ from healthy controls in this task, and these differences may be linked to altered sensitivity to reward and punishment [15,16,41,42]. Interestingly, alterations in decision making under ambiguity in patients with depression are positively associated with their symptom severity [12,14,38].

Studies on decision making under risk have reported inconsistent results. The *Game of Dice Task* (*GDT*) [43] is a computerized gambling task that has been used with different patient populations as well as healthy individuals to assess decision making under risk conditions [44,45,46,47,48]. In this task, participants have to maximize their capital within 18 trials. They are presented with different options associated with distinct gains/losses. Probabilities of events can be computed or estimated. In this task, information is explicitly given, and options remain stable over time. Lee et al. [21] and Fujino et al. [49] reported significant differences between schizophrenia patients and healthy individuals in the IGT but not in the GDT. Differently, Pedersen et al. [50] and Fond et al. [23] showed that, in the GDT, patients with schizophrenia make more frequent risky decisions and achieve a lower net-score (which is obtained by subtracting the number of disadvantageous/risky decisions from the number of advantageous/safe ones) compared to healthy individuals, although they visibly adjust their strategy over the course of the task [23,50]. Furthermore, a higher degree of positive schizophrenia symptomatology is correlated with a higher number of risky decisions, less use of negative feedback, and a lower GDT-net-score [50].

Affective disorders have been studied only sporadically in this context. A recent study has compared depressed patients who had attempted suicide within the last six months to depressed patients with no suicide attempts and a healthy control group on both the IGT and the GDT [13]. Results showed that the groups’ performances did not differ in ambiguous decision situations but clearly differed in risk decision situations. Depressed patients with a recent suicide attempt were significantly more likely to choose the risky options of the GDT than depressed patients without suicide attempts and healthy controls. Moreover, higher depression scores were associated with riskier choices [13]. However, this study did not include an assessment of general cognitive functioning [13]. For both patients with schizophrenia and patients with depression, it is not clear as to whether poor decision making under risk might be related to problems in other cognitive domains.

In this study, we used the PAG task to assess decision making under risk. In this task, participants have to maximize their capital within 40 trials by choosing between a safe option and a risky option (see below for details). In variance to the GDT, where the answer alternatives are stable over trials and long-term strategies can be applied, in the PAG task, the winning chance of the risky alternative and the type of safe alternative (gain 20€/pay 20€) change from trial to trial. Therefore, a good performance on this task requires not only accurate estimation of risk as in the GDT but also flexible adaptation to the changes in the decision situation, i.e., good executive functioning. In this study, participants also underwent a neuropsychological assessment tapping on executive function and memory.

On the basis of previous findings, we expected the patients with schizophrenia and the patients with major depression to perform more poorly than healthy controls on neuropsychological measures of executive function, verbal learning, and memory due to illness-related cognitive impairments [5,6,7,8,9,10,11,31]. Furthermore, we hypothesized that the patients’ cognitive performance correlates negatively with the severity of the clinical symptomology. That is, a severe degree of psychopathological symptoms is associated with a poorer cognitive performance. Given the frequently observed cognitive deficits in schizophrenia and depression on the one hand, and the involvement of executive function in decision making under risk on the other [43,44,45], we expected an association between the assessed cognitive abilities and the decision making performance on the PAG task. It may also be possible that both patient groups, in particular in relation to their poorer executive functioning, make more disadvantageous choices in the PAG task than healthy controls do and that they do not adapt their decision strategy to the changing decision conditions. We did not expect significant differences between the patient groups in the PAG task. To our knowledge, this is the first study comparing decision making under risk between patients with schizophrenia, patients with major depression, and healthy controls. Finally, we assumed that decision making under risk in patients with either schizophrenia or major depression is correlated with the degree of clinical symptoms, with the patients with a more severe symptomatology showing a poorer decision making performance [13,50].

## 2. Materials and Methods

### 2.1. Participants

We contacted 106 individuals to participate in this study (36 healthy individuals, 35 patients with schizophrenia, 35 patients with major depression). Six patients (five with schizophrenia, one with depression) refused to participate or subsequently decided to withdraw from the study. Additionally, two patients with schizophrenia and six patients with depression were excluded from the final sample because of either difficulties to understand the PAG task or a premorbid verbal intelligence quotient (IQ) <85 as measured by the *Mehrfach-Wahl-Wortschatz-Intelligenztest*, *Form B* (MWT-B; [51]). In the nonclinical group, six persons were excluded because of technical problems with the PAG task (two persons) or a history of neuropsychiatric disorders (four persons). The final sample of 86 participants, who completed the whole assessment protocol, included 28 patients with an *International Classification of Diseases*, version 10 (ICD-10; [52]) diagnosis of schizophrenia, 28 patients with an ICD-10 diagnosis of major depression, and 30 healthy controls with no history of substance abuse or other major medical, psychiatric, or neurological disorders. All participants had a premorbid verbal IQ of at least 85 (MWT-B; [51]).

All patients were inpatients consecutively recruited from two psychiatric hospitals in Austria. Diagnosis of either schizophrenia or major depression was made by a licensed psychiatrist according to the ICD-10 [52]. Exclusion criteria for patients with depression were present psychotic symptoms. Schizophrenia symptoms were rated using the *Positive and Negative Syndrome Scale* (*PANSS*) [53]. Depression severity was measured using the 21-item version of the *Hamilton Rating Scale for Depression* (*HAMD-21*) [54,55]. Both instruments were administered by the attending psychiatrist at the time of the examination. All patients received antipsychotic drugs or antidepressants, respectively.

The nonclinical group (healthy controls) was recruited through acquaintances or word of mouth. In the nonclinical group, people with major psychiatric disorders/history of major psychiatric disorders according to the Structured Clinical Interview for DSM-IV-Axis I Disorders (SCID screening) and people who reported having neurological diseases or using psychoactive medications were excluded from participation.

The study was conducted in accordance with the Declaration of Helsinki, and the protocol was approved by the ethics committee of the University of Graz (approval date 1 October 2015; code GZ. 39/84/63 ex 2014/15). After complete description of the study, all subjects provided written informed consent.

Every participant was tested in one session. The assessment generally lasted 80 to 90 min.

### 2.2. Neuropsychological Background Assessment

All participants were investigated with the *California Verbal Learning Test* (*CVLT*) [56] for the evaluation of episodic verbal learning and memory. The following measures of the CVLT were used as variables in the statistical analyses: The number of memorized words from trial 1 (immediate recall), the sum of memorized words from trials 1 to 5 (learning performance), the number of recalled words from trial 6 (short-delay free recall), trial 7 (long-delay free recall), and trial 8 (recognition correct = hits minus false positives). The *Trail Making Test Part A* (*TMT-A*) [57] was used to assess psychomotor speed, whereas the *Trail Making Test Part B* (*TMT-B*) was used to assess cognitive flexibility. For each part (A and B) the processing time (ln-transformed reaction times in s.) was used for statistical analyses. The *Regensburger word fluency test* (*RWT*) [58] was included gauging phonological and semantic verbal fluency. In the current study, the following two subtests of the RWT were used: The subtest “Animals”, which measures semantic-categorical verbal fluency, and the subtest “S-words”, which measures phonological verbal fluency. The total number of generated words in each subtest generated within two minutes was entered into the analyses. 

### 2.3. Assessment of Decision Making Abilities

#### Probability-Associated Gambling Task (PAG)

**Figure 1:**
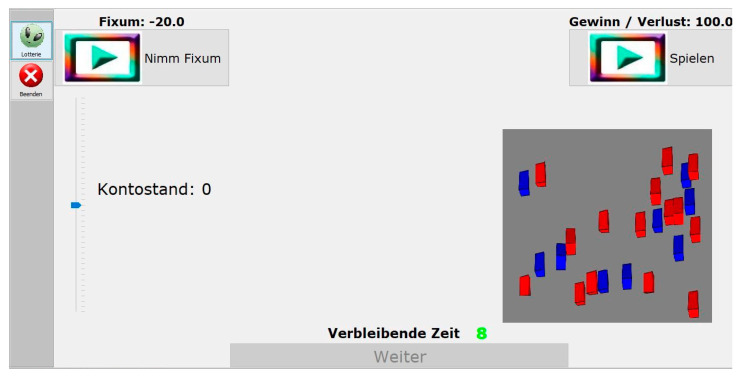
Probability-Associated Gambling Task (PAG) [59].

The PAG task [59] is a computer-based instrument for the measurement of decision making under risk. At the beginning of the task, the participant is instructed to imagine taking part in a lottery with the aim to win as much money as possible. The PAG task consists of 40 trials. In each trial the participant is required to choose one of two options within 10 s. The first option is a fixed sum (of either +20€ or −20€; for an example, Figure 1). The second option is the alternative to gamble. By choosing the latter, the participant has the possibility of winning or losing 100€, which depends on the winning probability. There are four different winning probabilities if the gambling alternative is chosen: Two low probabilities (3:21, *p* = 0.125; 9:15, *p* = 0.375) and two high probabilities (15:9, *p* = 0.625; 21:3, *p* = 0.875). The winning probabilities are visualized by 24 red and blue cubes shown in a grey-colored box in the right half of the screen. The ratio of red to blue cubes represents the winning probability. The red cubes are associated with winning, the blue ones with loss. Every probability occurs 10 times (five times each in combination with a negative fixed sum of −20€ or a positive fixed sum of +20€). Choosing the fixed sum alternative (±20€) is advantageous on trials of low winning probability (*p* = 0.125 and *p* = 0.375). The gamble alternative is advantageous on trials of high winning probability (*p* = 0.625 and *p* = 0.875). By choosing the alternative to gamble the computer mixes the cubes and draws one. If the drawn cube is red, the participant wins 100€, which are added to the personal account status (visualized by a scale in the left half of the screen; start capital = 0 Euro) and the sound of a cash drawer is played. If the drawn cube is blue, the participant loses 100€ from the personal account status, which is accompanied by a dull sound.

The following PAG variables are relevant for statistical data analyses:

- Arcsine-transformed frequency of gambles in each of the four winning probabilities in combination with a fixed sum of +20€;
- Arcsine-transformed frequency of gambles in each of the four winning probabilities in combination with a fixed sum of −20€;
- Ln-transformed total response time (RT) in ms;
- Total winning amount in €;
- Number of omission errors (trials where no alternative was chosen within the time limit of 10 s);
- Proportion of strategy changes from the fixed sum alternative to the gambling alternative when there is a positive change in the winning probability (from low to high);
- Proportion of strategy changes from the gambling alternative to the fixed sum alternative when there is a negative change in the winning probability (from high to low).

### 2.4. Statistical Analyses

A significance level of α = 0.05 (two-tailed) was used for all analyses. All analyses were conducted with the software (IBM SPSS Statistics for Windows, Version 26.0. Armonk, NY, USA: IBM Corp.).

*Demographical characteristics.* Demographical data of the patient groups (schizophrenia, depression) and the control group were compared by means of one-way analysis of variance (ANOVA) (age) or Pearson χ^2^ tests (sex, educational level).

*Neuropsychological background tests.* A multivariate analysis of variance (MANOVA) was performed with group (S, D, C) as between-subjects factor and cognitive test scores as dependent variables. Significant group differences were examined using one-way ANOVAs followed by post-hoc tests with Tukey’s HSD correction.

*PAG.* Arcsine-transformed frequencies of gambles were analyzed by means of a repeated-measures ANOVA with winning probability (*p* = 0.125, *p* = 0.375, *p* = 0.625, *p* = 0.875) and fixed sum (+20€, −20€) as within-subject factors and group (S, D, C) as between-subjects factor. A MANOVA was computed to assess group differences in ln-transformed mean response times (RTs), winning amount, and proportions of strategy change. The distribution of omission errors was compared between groups by means of a Pearson χ^2^ test.

*Correlation Analyses.* Pearson’s correlation analyses were carried out for the three groups separately. We were interested in the associations between clinical variables (PANSS, HAMD-21) and neuropsychological test scores, between clinical variables and decision making performance, as well as between decision making performance and neuropsychological test scores. For the latter, group comparisons (schizophrenia vs. healthy controls, depression vs. healthy controls) were then performed on Fisher’s z-transformed correlation coefficients (http://vassarstats.net/rdiff.html; accessed on 31 August 2021). In order to reduce the number of variables, we focused on the frequency of gambles in the *p* = 0.125 condition, response times, and the proportions of strategy changes in case of a positive change in the winning probability. These were the decision making variables where significant group differences emerged.

## 3. Results

### 3.1. Sociodemographic and Clinical Data

The sociodemographic and clinical data of the participant groups are displayed in Table 1. The three groups did not differ significantly in terms of age, sex, and level of education (all *p*_s_ > 0.1).

### 3.2. Neuropsychological Background Scores

A MANOVA carried out on cognitive test scores with group (S, D, C) as between-subjects factor was significant (F_18,152_ = 3.867, *p* < 0.001, η_p_**^2^** = 0.314). A significant group effect emerged in all variables (*p*_s_ < 0.01). Post-hoc contrasts with Tukey’s HSD correction indicated that all three groups differed from each other in measures of immediate recall (CVLT trial 1), learning (CVLT trials 1-to-5), and phonological verbal fluency (S-words/2 min; Table 2) with the control group performing better than both patient groups (*p*_s_ < 0.05). In turn, patients with depression performed better than patients with schizophrenia (*p*_s_ < 0.05). In other measures of memory (trial 6 short-delay free recall, trial 7 long-delay free recall, recognition correct) as well as in semantic-categorical verbal fluency (animals/2 min), psychomotor speed (TMT-A), and cognitive flexibility (TMT-B), patient groups did not differ from each other (*p*_s_ > 0.05), but both scored lower than healthy controls (*p*_s_ < 0.05). We obtained very similar results when cognitive scores were analyzed by means of non-parametric methods (Kruskal-Wallis tests). In sum, in several neuropsychological measures of verbal memory and executive function patient groups showed very similar performances, whereas both scored more poorly than healthy controls.

### 3.3. Decision Making Performance

**Figure 2:**
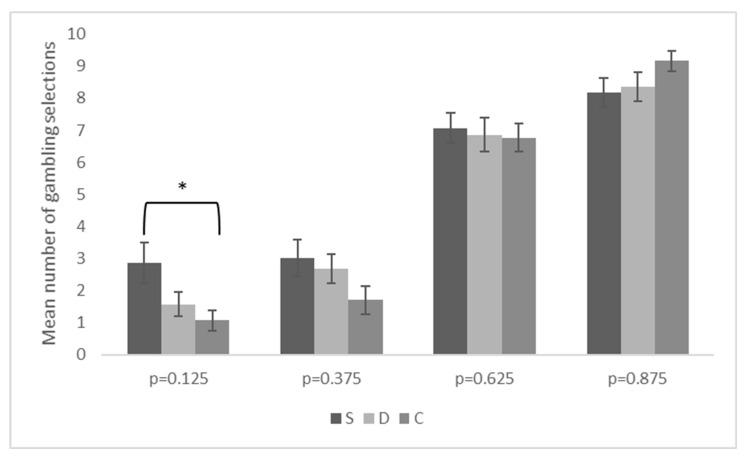
Mean number of gambling selections in the PAG task as a function of winning probability and group. Legend: S = patients with schizophrenia; D = patients with major depression; C = healthy controls; p = PAG winning probability; * indicates a significant group difference, *p* < 0.05. Error bars indicate the standard error of the mean.

A repeated-measures ANOVA conducted on the arcsine-transformed frequency of gambles indicated a significant main effect of winning probability (F_3,249_ = 180.655, *p* < 0.001, η_p_**^2^** = 0.685), a significant main effect of fixed sum (F_1,83_ = 15.236, *p* < 0.001, η_p_**^2^** = 0.155), and a significant interaction between winning probability and group (F_6,249_ = 2.294, *p* < 0.01, η_p_**^2^** = 0.052). Other results were not significant. In general, participants showed sensitivity to the winning probability by gambling increasingly more frequently as the winning probability increased. Moreover, the gambling alternative was chosen more often when the fixed sum alternative was −20€ than when it was +20€. The significant two-way interaction was further explored by analyzing group differences in each winning probability by means of one-way ANOVAs. Post-hoc contrasts were performed with Tukey’s HSD correction. As shown in Figure 2, patients with schizophrenia differed significantly from controls at the lowest winning probability by selecting the gambling alternative more frequently than healthy participants did (*p* < 0.05). Other contrasts were not significant.

Furthermore, a MANOVA performed on the ln-transformed RTs, winning amount, and proportions of strategy changes was significant (F_8,162_ = 3.665, *p* = 0.001, η_p_**^2^** = 0.153). Significant group differences emerged for RTs (*p* < 0.001) and the proportion of strategy changes in case of a positive change in the winning probability (*p* < 0.05), but not for the winning amount and the proportion of strategy changes in case of a negative change in the winning probability (Table 3). Post-hoc contrasts with Tukey’s HSD correction indicated that both patient groups responded more slowly than controls but did not differ from each other in terms of RTs. Furthermore, patients with schizophrenia made fewer strategy changes than controls. Other differences were not significant. Very similar results were found when using non-parametric methods (Kruskal-Wallis tests).

Participants committing at least one omission error were as follow: Three controls (10%), 15 patients with schizophrenia (53.57%), and 10 patients with depression (35.71%; Pearson χ^2^ _2,86_ = 12.71, *p* < 0.001). As indicated by pairwise χ^2^ tests, patient distributions did not differ from each other but both differed from the control distribution (*p*_s_ < 0.05). In sum, there were more patients with schizophrenia or depression committing an omission error than controls. This is congruent with the finding that patient groups responded generally more slowly than healthy controls did.

### 3.4. Correlations between Clinical Variables and Neuropsychological Background Scores

For patients with schizophrenia, there were significant correlations between PANSS negative symptoms scores and semantic-categorical verbal fluency (r = −0.530, *p* < 0.01) as well as between PANSS general psychopathology scores and cognitive flexibility (r = 0.409, *p* < 0.05). No significant correlations were found between severity of symptomatology and measures of memory. In general, schizophrenia patients with a more pronounced symptomatology performed more poorly in measures of executive function. For patients with depression, we found no significant correlations between clinical variables and neuropsychological background scores.

### 3.5. Correlations between Clinical Variables and Decision Making Performance

We found no significant correlations for patients with depression. For patients with schizophrenia, there was a significant correlation between the proportion of strategy changes in case of a positive change in winning probability and PANSS general symptomatology scores (*r* = −0.377, *p* < 0.05). Similarly, the proportion of strategy changes in case of a positive change in winning probability correlated significantly with PANSS negative symptoms scores (*r* = −0.391, *p* < 0.01). Other correlations were not significant. In sum, schizophrenia patients with a more pronounced symptomatology made fewer strategy changes.

### 3.6. Correlations between Neuropsychological Background Scores and Decision Making Performance

Results of correlation analyses for the three groups separately are displayed in Table 4, Table 5 and Table 6. There were significant associations between decision making and neuropsychological background scores. Patients with schizophrenia or depression who obtained higher scores in memory and executive function tests performed better in the PAG task. Healthy controls with better long-term memory scores made slower decisions. Also, those being slower in a neuropsychological test assessing psychomotor speed made more advantageous decisions and a higher proportion of strategy changes.

Results of the comparison of the Fisher’s z-transformed correlation coefficients between healthy controls and patient groups can be found in the Supplementary Material Tables S1 and S2. In this regard, we found no significant group differences.

## 4. Discussion

The main purposes of this study were to investigate (1) whether there are differences in decision making under risk between patients with schizophrenia, patients with depression, and healthy controls, and (2) which impact cognitive deficits and clinical factors have on decision making behavior under risk in schizophrenia and depression.

### 4.1. Cognitive Performance

Results of the current study revealed that patients suffering from schizophrenia or depression performed more poorly than healthy controls in all cognitive measures administered. Specifically, reduced performances in verbal memory and learning, semantic-categorical and phonological verbal fluency, psychomotor speed, and cognitive flexibility were demonstrated in patients with schizophrenia and patients with depression compared to healthy individuals. These findings are in line with results of previous studies [8,23,31,32,33,34,35,36]. Interestingly, we also found significant differences in some cognitive domains between the two patient groups. Schizophrenia patients achieved lower scores than depressed patients in learning and immediate memory as well as in phonological verbal fluency. A trend towards significance was also evident in cognitive flexibility. These group differences have already been reported in previous research on this topic [60].

In this study, we could not find a significant association between the patients’ severity of depression symptoms and their cognitive scores. This is in contrast with results of a meta-analysis by McDermott and Ebmeier [7], which showed a negative correlation of depression severity with multiple cognitive parameters. A lack of a significant correlation between depression severity and cognition may be due to the comparatively low degree of depressive symptoms in our patients. It should be also noted that the age range of our depressive patients is relatively large (18 to 71 y.o.). Therefore, it is possible that the disease duration as well as the time of the disease onset (which were not recorded for the purposes of this study) varies notably within our group of depressive patients. These are factors that may influence both expression of the clinical symptoms and the cognitive performances of the patients.

Our expectation of an association between symptomatology severity and cognitive performances was partially validated for patients with schizophrenia. In this study, we found that a more pronounced negative symptomatology is related to poorer scores in a semantic-categorical verbal fluency test, suggesting that negative symptoms are predominantly associated with cognitive deficits. In our study, patients with schizophrenia rated negative symptoms (PANSS *M* = 23.11) higher than positive symptoms (PANSS *M* = 17.96), which is partly due to the fact that the patients were treated with antipsychotic medication to reduce positive symptoms. We also found a significant negative correlation between general psychopathology of schizophrenia and cognitive flexibility. In sum, a more pronounced negative and general psychopathological symptomatology is associated with poorer executive function in patients with schizophrenia.

### 4.2. Decision Making under Risk

In the PAG task, both patient groups made significantly more omission errors (missed trials) and showed longer response times than the healthy control group, which may be linked—as indicated by results of a correlation analysis—to reduced psychomotor speed and cognitive flexibility. Furthermore, we found that patients with schizophrenia chose the gambling alternative in the lowest winning probability more often than healthy controls did. This can be considered as a risky, disadvantageous decision behavior. Relative to healthy controls, patients with schizophrenia also made fewer strategy changes in accordance to a positive change in the winning probability, demonstrating less flexibility in adapting their decision behavior to the changes in the decision situation. In sum, our results indicate that the decision making behavior under risk of schizophrenia patients is characterized by risky, disadvantageous choices and by reduced flexibility. This confirms and extends previous research by Pedersen et al. [50] and Fond et al. [23], who reported abnormalities in decision making under risk for patients with schizophrenia by using the GDT. Differently from the PAG task, the GDT is a decision making task with stable conditions. Here, participants can develop long-term decision strategies. In the PAG task, participants have to flexibly and quickly adapt their decision behavior to the changing conditions as well as estimate the winning probabilities. Patients with schizophrenia show therefore difficulties in both gambling tasks.

Results of a correlation analysis further suggest for the patients with schizophrenia a negative association between changes in decision strategies and the severity of negative and general psychopathological symptomatology. This is in line with previous studies also showing abnormalities in the decision making under risk pattern of patients with schizophrenia to be related with a more pronounced symptomatology [50]. For example, Pedersen et al. [50] showed that a higher degree of positive symptoms is correlated with riskier choices in schizophrenia patients. In this study, we also found that the number of choices in the lowest winning probability is associated with the patients’ learning performance in a verbal memory test. It seems plausible that patients with schizophrenia with cognitive deficits and stronger psychopathological symptomatology decide less advantageously in the PAG task and show less flexibility in adapting their strategies to the changing decision conditions. In our study, patients with schizophrenia performed more poorly in different memory and executive function measures even in comparison to patients with depression. This may, at least partly, explain why patients with schizophrenia differed from healthy controls in different decision making under risk measures, while patients with depression did not.

In the PAG task, patients with depression did not significantly differ from healthy controls and from patients with schizophrenia in terms of choices, strategy changes, and total winning amount. We only found that, similarly to patients with schizophrenia, patients with depression responded more slowly than healthy controls, which suggest that both patient groups did not make impulsive decisions. Results of a correlation analysis indicated no association between the decision making performance and the severity of depression symptoms, which is in contrast with a previous study by Deisenhammer et al. [13]. It should be, however, noted that, regarding depression severity, our patient group is similar to the group of depressed patients without suicide attempts reported by Deisenhammer et al. [13], who did not differ from healthy controls in the GDT. Deisenhammer et al. [13] reported poorer decision making behavior under risk for severely depressed patients who had recently attempted suicide but not for depressed patients without suicide attempts. Therefore, it seems possible that difficulties in decision making under risk may be evident in those patients who have severe depression. In our study, we also found an association between decision times and different measures of memory and executive functions (immediate recall, psychomotor speed, cognitive flexibility, phonological verbal fluency). In general, longer decision times correlated with a poorer cognitive performance in depressed patients. These results suggest that, despite being slow and having cognitive deficits, patients with depression can make advantageous decisions under risk and flexibly adapt their strategies to the changing decision conditions of the PAG task.

In this study, a better decision making performance of patients with schizophrenia or depression was associated with better memory and executive function scores. For healthy controls, we found that slower decisions were associated with better long-term memory scores. Also, more advantageous decisions and a higher proportion of strategy changes were associated with slower response times in a neuropsychological test assessing psychomotor speed. A direct comparison of the correlation coefficients between groups failed to reach significance. Although these results might seem contradicting, at a first glance, it should be noted that performance in decision making tasks may load on memory and executive functions differently depending on whether an individual has cognitive deficits or not. Cognitive impairments in attention, memory, and executive function are to be expected in patients with schizophrenia or depression (but not in healthy individuals) and may relevantly influence the patients’ ability to make flexible and advantageous decisions under risk. In other words, memory and executive function may support differently the decision making performance of clinical and nonclinical individuals.

In general, all three groups showed a similar decision pattern in the PAG task: the higher the winning probability, the more often the gambling alternative was chosen. Moreover, participants chose the gambling alternative more often in case of a fixed sum alternative of −20€ than in case of a fixed sum alternative of +€20. This result is in accordance with those of previous studies [13,21,49,59] and implies that, across all groups, there was a basic understanding of the decision task and that the decision behavior was modulated by the winning probability and the fixed sum alternative. With the exception of the patients with schizophrenia, who made relatively more risky choices than healthy controls with the lowest winning probability, patients with depression similarly to healthy individuals tended to avoid making frequent risky decisions that would have resulted in high losses.

In line with previous research [43,44,45], results of this study on schizophrenia and depression point to the importance of memory and executive function in decision making under risk. Disease-related cognitive impairments may—at least to some extent—adversely affect the decision making behavior under risk of patients with schizophrenia or depression. Severity of clinical symptoms also seem to play a role both in cognitive functioning and in the decision making behavior of patients with schizophrenia. As regard patients with depression, we did not find correlations between depression symptoms and measures of memory, executive functions, and decision making. This is possibly because of a low disease severity and variance in our sample. Together with previous studies [13,50], our results, in general, suggest that patients with more pronounced psychopathological symptoms may have cognitive deficits (e.g., in executive function) and problems in making advantageous decisions under risk. Therefore, successful treatment of psychopathological symptoms and reduction of the disease severity may have positive effects on decision making under risk as well as on the cognitive performance of patients with schizophrenia or depression. We also found that both patients with depression and patients with schizophrenia need more time in making decisions under risk. These findings are of relevance in the treatment and general management of these patient groups. They suggest that, for example, in the medical setting, persons with depression or schizophrenia may need additional time or assistance to make decisions concerning psychopharmacological treatment. This could have a positive effect on the patients’ willingness to undergo drug treatment and on their adherence to therapy plans.

### 4.3. Limitations

We acknowledge some limitations of this study. First, at the time of the investigation, all patients had an inpatient status and received antidepressant/antipsychotic drug therapy. We cannot therefore exclude possible effects of psychopharmaceutic drug intake on cognition, although indications about the direction of these effects are quite controversial in the literature [61,62,63], and current pharmacotherapy options to treat cognitive dysfunction in depressed patients or patients with schizophrenia remain extremely limited and poorly studied [64]. Second, schizophrenia and depression are syndromes characterized by a great heterogeneity, which is expressed in terms of their symptomatic manifestations, course of illness, regularity of phases, comorbidities, and also duration of illness. In this study, we did not control for all these factors and, therefore, can only cautiously make conclusions about the influence of clinical characteristics on cognition and decision making under risk of patients with schizophrenia or depression. Also, we did not assess depressive symptoms in patients with schizophrenia. Previous research have suggested an overlap between depression and negative symptoms in schizophrenia [65]. In a systematic review, Krynicki et al. [66] proposed a dimensional model to explain the relationship between these two domains and highlighted the importance of employing appropriate scales to distinguish depressive features from negative symptoms in schizophrenia. We did not also administer the PANSS or the HAMD-21 to the nonclinical group and, therefore, may have missed possible subclinical symptoms in the healthy control group. Future studies should clarify comorbidities and subclinical symptoms diagnostically more precisely in order to exclude interfering factors even more strictly. Third, it has been demonstrated that the performance of patients with schizophrenia in different cognitive domains is positively correlated with their motivation and willingness to exert effort [67,68]. Although patients participated voluntarily in this study and did not show evident signs of demotivation or tiredness, we did not actively control for these factors during the investigation (e.g., through a visual analogue scale for the self-estimation of motivation or tiredness). Finally, individuals may use different strategies when making decisions at risk. The actual implementation of the PAG task does not allow for inquiring participants on an item-by-item basis for the applied strategies. This may be of interest also in order to exclude possible arbitrary decisions in a fictitious decision making situation with no real harmful consequences for the participant. Future fMRI studies may also help uncover which brain structures and networks are associated with advantageous and disadvantageous decisions in the PAG task in patients with schizophrenia or depression.

## 5. Conclusions

Our study compares patients with schizophrenia, patients with depression, and healthy peers in a task assessing decision making under risk. It also investigates possible relations between decision making, cognition, and psychopathological severity. Results show that patients with depression or schizophrenia may present with deficits in different memory and executive function measures. Apart from slow responses, patients with depression do not significantly differ from healthy individuals in a decision making under risk task requiring flexible adaptation of the decision behavior to changes in the decision situation. Differently, patients with schizophrenia decide more slowly, riskier, and less flexibly than healthy controls do. For them, the decision making behavior correlates with the severity of the psychopathological symptomatology. In both groups, results point to the possible influence of memory and executive function impairments in decision making under risk. Together with previous studies [13,23,50], our findings suggest that patients with schizophrenia or depression may have difficulties under risk conditions when quick and flexible decisions are required and that these difficulties may be more pronounced in those patients with marked cognitive deficits or severe clinical symptoms.

## Figures and Tables

**Table 1:** Sociodemographic and clinical data.

	Participant Groups		
	S (n = 28)	D (n = 28)	C (n = 30)
Age (M, SD)	42.57 (12.26)	42.79 (15.29)	42.40 (15.28)
Sex			
- Male	15	12	11
- Female	13	16	19
Education			
- 1: Less than high school	22	19	20
- 2: High school degree	4	9	7
- 3: Some college	2	0	3
Depression severity (M, SD) [HAMD-21 score]	-	18.11 (5.92)	-
Schizophrenia symptomatology (M, SD) [PANSS score]			
- Positive symptoms	17.96 (5.70)	-	-
- Negative symptoms	23.11 (7.76)	-	-
- General psychopathology	37.79 (9.73)	-	-

M = mean; SD = standard deviation; S = patients with schizophrenia; D = patients with major depression; C = healthy controls; PANSS = Positive and Negative Syndrome Scale; HAMD-21 = Hamilton Rating Scale for Depression, 21-item version. The level of education was measured in terms of highest level of education completed.

**Table 2:** Performance in neuropsychological background tests.

	Participant Groups		Group Comparisons			
Neuropsychological Parameters	S (n = 28)	D (n = 28)	C (n = 30)	S vs. D (*p*-Value)	S vs. C (*p*-Value)	D vs. C (*p*-Value)
CVLT [correctly recalled items] (M, SD)						
- learning (trials 1–5)	35.71 (12.47)	44.00 (12.00)	54.53 (8.66)	**0.018**	**0.000**	**0.002**
- immediate memory (trial 1)	4.39 (1.50)	6.14 (2.17)	7.53 (1.81)	**0.002**	**0.000**	**0.015**
- short-delay free recall (trial 6)	7.04 (3.23)	8.21 (3.75)	11.87 (2.49)	0.352	**0.000**	**0.000**
- long-delay free recall (trial 7)	6.43 (3.51)	8.39 (4.31)	11.17 (3.77)	0.146	**0.000**	**0.021**
- recognition corrected	10.21 (3.63)	11.25 (3.92)	13.60 (1.69)	0.452	**0.000**	**0.018**
TMT [s, ln-transformed] (M, SD)						
- psychomotor speed (Part A)	51.89 (20.00)	56.29 (41.89)	34.37 (14.52)	0.926	**0.003**	**0.009**
- cognitive flexibility (Part B)	149.43 (67.14)	122.43 (67.77)	65.57 (20.49)	0.081	**0.000**	**0.000**
RWT [number of produced words] (M, SD)						
- Semantic-categorical verbal fluency (“Animals”/2 min)	27.64 (8.82)	30.21 (9.02)	37.97 (8.23)	0.512	**0.000**	**0.003**
- Phonological verbal fluency (“S-words”/2 min)	17.29 (5.66)	23.00 (8.81)	29.80 (7.45)	**0.014**	**0.000**	**0.002**

M = mean; SD = standard deviation; S = patients with schizophrenia; D = patients with major depression; C = healthy controls; CVLT = California Verbal Learning Test; TMT = Trail Making Test; RWT = Regensburger word fluency test; Post-hoc contrasts were performed with Tukey’s HSD correction; bold values indicate significant group differences, *p* < 0.05.

**Table 3:** Response times, winning amount, and strategy changes in the PAG task.

	Participant Groups			Group Comparisons		
PAG Parameters (M, SD)	S (n = 28)	D (n = 28)	C (n = 30)	S vs. D (*p*-Value)	S vs. C (*p*-Value)	D vs. C (*p*-Value)
Response time [ms, ln-transformed]	4064.15 (1477.69)	3684.78 (1281.18)	2451.21 (1026.67)	0.646	**0.000**	**0.001**
Total winning amount [€]	530.00 (535.45)	655.00 (494.01)	805.33 (409.40)	0.596	0.081	0.463
Strategy Changes [proportions]						
- From fixed sum to gambling at a positive change of winning probability	0.57 (0.32)	0.60 (0.30)	0.75 (0.25)	0.886	**0.043**	0.125
- From gambling to fixed sum at a negative change of winning probability	0.55 (0.31)	0.61 (0.33)	0.69 (0.25)	0.747	0.175	0.541

PAG = Probability-Associated Gambling Task; M = mean; SD = standard deviation; S = patients with schizophrenia; D = patients with major depression; C = healthy controls; bold values indicate significant group differences, *p* < 0.05.

**Table 4:** Pearson’s correlations between decision making performance and neuropsychological background scores in patients with schizophrenia (n = 28).

	PAG Task Parameters					
	Frequency of Gambles with *p* = 0.125		Total Response Times		Proportion of Strategy Changes	
Neuropsychological Parameters	*r*	*p*	*r*	*p*	*r*	*p*
CVLT [correctly recalled items]						
- learning (trials 1–5)	**−0.407**	**0.032**	−0.334	0.082	0.285	0.142
- immediate memory (trial 1)	−0.018	0.929	−0.210	0.283	−0.027	0.893
- short-delay free recall (trial 6)	−0.270	0.165	−0.292	0.131	0.155	0.432
- long-delay free recall (trial 7)	−0.102	0.604	−0.301	0.119	−0.090	0.650
- recognition corrected	−0.166	0.398	−0.305	0.115	0.007	0.972
TMT [s]						
- psychomotor speed (Part A)	0.235	0.229	**0.412**	**0.030**	−0.192	0.328
- cognitive flexibility (Part B)	0.255	0.190	**0.448**	**0.017**	−0.341	0.076
RWT [number of produced words]						
- semantic-categorial verbal fluency (“Animals”/2 min)	−0.352	0.067	−0.103	0.602	0.328	0.089
- phonological verbal fluency (“S-words”/2 min)	−0.344	0.073	−0.276	0.155	0.226	0.248

PAG = Probability Associated Gambling Task; RT measured in ms. and ln-transformed; CVLT = California Verbal Learning Test; TMT = Trail Making Test; RWT = Regensburger word fluency test; bold values indicate significant correlations, *p* < 0.05.

**Table 5:** Pearson’s correlations between decision making performance and neuropsychological background scores in patients with major depression (n = 28).

	PAG Task Parameters					
	Frequency of Gambles with *p* = 0.125		Total Response Times		Proportion of Strategy Changes	
Neuropsychological Parameters	*r*	*p*	*r*	*p*	*r*	*p*
CVLT [correctly recalled items]						
- learning (trials 1–5)	0.030	0.880	−0.159	0.418	−0.223	0.255
- immediate memory (trial 1)	−0.051	0.797	**−0.383**	**0.044**	−0.201	0.305
- short-delay free recall (trial 6)	−0.095	0.632	−0.154	0.434	−0.129	0.512
- long-delay free recall (trial 7)	−0.019	0.925	−0.186	0.342	−0.178	0.365
- recognition corrected	−0.140	0.477	−0.273	0.160	−0.127	0.520
TMT [s]						
- psychomotor speed (Part A)	−0.034	0.865	**0.510**	**0.006**	0.220	0.261
- cognitive flexibility (Part B)	0.117	0.554	**0.511**	**0.005**	−0.060	0.761
RWT [number of produced words]						
- semantic-categorial verbal fluency (“Animals”/2 min)	−0.229	0.241	−0.044	0.825	0.172	0.383
- phonological verbal fluency (“S-words”/2 min)	−0.338	0.078	**−0.468**	**0.012**	0.303	0.117

PAG = Probability Associated Gambling Task; RT measured in ms. and ln-transformed; CVLT = California Verbal Learning Test; TMT = Trail Making Test; RWT = Regensburger word fluency test; bold values indicate significant corre-lations, *p* < 0.05.

**Table 6:** Pearson’s correlations between decision making performance and neuropsychological background scores in healthy controls (n = 30).

	PAG Task Parameters					
	Frequency of Gambles with *p* = 0.125		Total Response Times		Proportion of Strategy Changes	
Neuropsychological Parameters	*r*	*p*	*r*	*p*	*r*	*p*
CVLT [correctly recalled items]						
- learning (trials 1–5)	0.110	0.564	−0.009	0.963	−0.196	0.299
- immediate memory (trial 1)	−0.033	0.861	−0.011	0.953	−0.032	0.866
- short-delay free recall (trial 6)	0.249	0.185	0.271	0.147	−0.162	0.394
- long-delay free recall (trial 7)	0.271	0.147	**0.367**	**0.046**	−0.191	0.311
- recognition corrected	0.115	0.546	0.131	0.490	−0.148	0.436
TMT [s]						
- psychomotor speed (Part A)	**−0.448**	**0.013**	−0.083	0.662	**0.465**	**0.010**
- cognitive flexibility (Part B)	−0.069	0.719	0.136	0.472	0.026	0.890
RWT [number of produced words]						
- semantic-categorial verbal fluency (“Animals”/2 min)	0.149	0.431	0.195	0.301	−0.060	0.755
- phonological verbal fluency (“S-words”/2 min)	−0.012	0.949	0.007	0.970	0.038	0.843

PAG = Probability Associated Gambling Task; RT measured in ms. and ln-transformed; CVLT = California Verbal Learning Test; TMT = Trail Making Test; RWT = Regensburger word fluency test; bold values indicate significant correlations, *p* < 0.05.

## Data Availability

The datasets generated during and/or analyzed during the current study are available from the corresponding author on reasonable request.

## Supplementary Materials

The following are available online at https://www.mdpi.com/article/10.3390/brainsci11091178/s1, Table S1: Fisher’s-z-transformations for the comparison of the Pearson’s correlation coefficients between patients with schizophrenia (n = 28) and healthy controls (n = 30), Table S2: Fisher’s-z-transformations for the comparison of the Pearson’s correlation coefficients between patients with major depression (n = 28) and healthy controls (n = 30).
