# Supplementary material for: Decision Making under Risk in Patients Suffering from Schizophrenia or Depression
Source: Brain Sci. 2021 Sep 7;11(9):1178. doi: 10.3390/brainsci11091178 (PMC8470442; doi:10.3390/brainsci11091178)
Supplement: Supplementary file 1 [file brainsci-11-01178-s001.zip › brainsci-1345738-supplementary.pdf]

Table S1. Fisher's-z-transformations for the comparison of the Pearson's correlation coefficients between patients with schizophrenia (n=28) and healthy controls (n=30).

	PAG task parameters					
	Frequency of gambles with p=0.125		Total response times		Proportion of strategy changes	
**Neuropsychological parameters**	*z*	*p*	*z*	*p*	*z*	*p*
**CVLT** [correctly recalled items]						
- learning (trials 1-5)	1.16	.246	1.22	.222	0.34	.734
- immediate memory (trial 1)	-0.05	.960	0.73	.465	0.02	.984
- short-delay free recall (trial 6)	0.08	.936	0.08	.936	-0.03	.976
- long-delay free recall (trial 7)	-0.63	.529	-0.27	.787	-0.37	.711
- recognition corrected	0.19	.849	0.66	.509	-0.51	.610
**TMT** [sec]						
- psychomotor speed (Part A)	-0.87	.384	1.28	.200	-1.11	.267
- cognitive flexibility (Part B)	0.69	.490	1.24	.215	1.19	.234
**RWT** [number of produced words]						
- semantic-categorial verbal fluency ("Animals"/2 min)	0.78	.435	-0.34	.734	1.01	.312
- phonological verbal fluency ("S-words"/2 min)	1.25	.211	1.00	.317	0.69	.490

PAG = Probability Associated Gambling Task; RT measured in msec. and ln-transformed; CVLT = California Verbal Learning Test; TMT = Trail Making Test; RWT = Regensburger word fluency test; bold values indicate significant correlations, $p < .05$, two-tailed.

Table S2. Fisher's-z-transformations for the comparison of the Pearson's correlation coefficients between patients with major depression (n=28) and healthy controls (n=30).

Neuropsychological parameters	PAG task parameters					
	Frequency of gambles with p=0.125		Total response times		Proportion of strategy changes	
	z	*p*	*z*	*p*	*z*	*p*
**CVLT** [correctly recalled items]						
- learning (trials 1-5)	-0.29	.772	0.55	.582	0.10	.920
- immediate memory (trial 1)	0.06	.952	1.41	.158	0.62	.535
- short-delay free recall (trial 6)	-0.57	.569	-0.44	.660	-0.12	.904
- long-delay free recall (trial 7)	-0.93	.352	-0.71	.478	-0.05	.960
- recognition corrected	0.09	.928	0.53	.593	-0.08	.936
**TMT** [sec]						
- psychomotor speed (Part A)	-1.61	.107	1.73	.084	-1.01	.312
- cognitive flexibility (Part B)	0.17	.865	1.54	.124	0.12	.904
**RWT** [number of produced words]						
- semantic-categorial verbal fluency ("Animals"/2 min)	0.30	.764	-0.55	.582	0.41	.682
- phonological verbal fluency ("S-words"/2 min)	1.22	.222	1.80	.072	0.99	.322

PAG = Probability Associated Gambling Task; RT measured in msec. and ln-transformed; CVLT = California Verbal Learning Test; TMT = Trail Making Test; RWT = Regensburger word fluency test; bold values indicate significant correlations, $p < .05$, two-tailed.
